# *Lactiplantibacillus plantarum* P470 Isolated from Fermented Chinese Chives Has the Potential to Improve In Vitro the Intestinal Microbiota and Biological Activity in Feces of Coronary Heart Disease (CHD) Patients

**DOI:** 10.3390/nu16172945

**Published:** 2024-09-02

**Authors:** Lingshuang Yang, Yuwei Wu, Juan Yang, Ying Li, Xinyu Zhao, Tingting Liang, Longyan Li, Tong Jiang, Tiantian Zhang, Jumei Zhang, Haojie Zhong, Xinqiang Xie, Qingping Wu

**Affiliations:** 1 College of Food Science, South China Agricultural University, Guangzhou 510642, China; yangls8272@163.com; 2 National Health Commission Science and Technology Innovation Platform for Nutrition and Safety of Microbial Food, Guangdong Provincial Key Laboratory of Microbial Safety and Health, State Key Laboratory of Applied Microbiology Southern China, Institute of Microbiology, Guangdong Academy of Sciences, Guangzhou 510070, China; 13503036950@163.com (Y.W.); liying@gdim.cn (Y.L.); zhaoxy9897@163.com (X.Z.); liangtingting@gdph.org.cn (T.L.); 18868006204@163.com (L.L.); jt0925@stu.scau.edu.cn (T.J.); ttsghww@163.com (T.Z.); zhangjm926@126.com (J.Z.); 3 The First Affiliated Hospital, School of Clinical Medicine of Guangdong Pharmaceutical University, Guangzhou 510060, China; juanayangon@163.com (J.Y.); jaxzhong@126.com (H.Z.)

**Keywords:** *Lactiplantibacillus plantarum* P470, fermented Chinese chives, intestinal microbiota, biological activity, coronary heart disease (CHD)

## Abstract

Traditional fermented foods are known to offer cardiovascular health benefits. However, the potential of fermented Chinese chives (FCC) in reducing coronary heart disease (CHD) remains unclear. This study employed anaerobic fermentation to investigate *Lactiplantibacillus plantarum* (*L. plantarum*) P470 from FCC. The results indicated that *L. plantarum* P470 enhanced hydroxyl radical scavenging and exhibited anti-inflammatory effects on RAW264.7 macrophages in the fecal fermentation supernatant of CHD patients. These effects were attributed to the modulation of gut microbiota and metabolites, including short-chain fatty acids (SCFAs). Specifically, *L. plantarum* P470 increased the abundance of *Bacteroides* and *Lactobacillus* while decreasing *Escherichia-Shigella*, *Enterobacter*, *Veillonella*, *Eggerthella*, and *Helicobacter* in CHD patient fecal samples. Furthermore, *L. plantarum* P470 regulated the biosynthesis of unsaturated fatty acids and linoleic acid metabolism. These findings suggest that *L. plantarum* P470 from FCC can improve the fecal physiological status in patients with CHD by modulating intestinal microbiota, promoting SCFA production, and regulating lipid metabolism.

## 1. Introduction

Coronary heart disease (CHD) with atherosclerosis is a persistent inflammatory and oxidative process that remains a major contributor to illness and death on a global scale [1]. Atherosclerosis leads to the accumulation of cholesterol carried by low-density lipoproteins (LDLs) within the arterial wall. The oxidative modification of LDL particles in the blood vessels is a key mechanism that initiates an inflammatory response, damaging the inner lining of the vessels [2]. In response to this inflammation, macrophages are attracted to the inflammatory sites and engulf the modified LDL particles, forming lipid-rich foam cells. These foam cells exacerbate local inflammation by releasing pro-inflammatory mediators such as cytokines like interleukin (IL)-1β and tumor necrosis factor (TNF)-α. These primary pro-inflammatory cytokines are released at every stage of atherosclerotic plaque formation and trigger the production of secondary signaling cytokines such as IL-6 [3]. Lipopolysaccharide (LPS) is a compound found in the cell walls of Gram-negative bacteria that plays a crucial role in inducing inflammatory responses and contributing to various inflammatory diseases. It can drive macrophages to polarize into the M1 phenotype, exacerbating inflammation and ultimately leading to the worsening of atherosclerosis (AS) [4].

Traditionally, CHD with atherosclerosis has been treated with medications, such as statins [5], anticoagulants (NOAC) [6], and adrenoceptor-blocking agents [6]. However, these treatments may cause adverse effects, including gastrointestinal issues, fatigue, headaches, scalp tingling, skin rashes, urinary retention, and erectile dysfunction [7,8]. Therefore, exploring novel strategies and therapies for the prevention and treatment of CHD is necessary. Patients with atherosclerosis often exhibit intestinal dysbiosis, suggesting that modulating the intestinal microbiota could offer a novel method for improving CHD management. Karlsson (2012) et al. proposed that, in patients with atherosclerosis, Collinsella levels in the gut microbiota are significantly higher, whereas levels of *Roseburia* and *Eubacterium* are markedly lower. Additionally, the composition of the intestinal metagenome is closely associated with the host’s inflammatory condition [9]. In a separate study, the prevalence of *Prevotella* was notably higher, while levels of *Clostridium* and *Faecalibacterium* were significantly reduced in male patients with atherosclerosis in Poland [10]. In Chinese male subjects, the presence of Streptococcus and Escherichia was markedly elevated, while Bacteroides and Prevotella were notably diminished [10]. This underscores the potential importance of exploring new treatment approaches that involve regulating the gut microbiota to prevent or treat CHD.

Recently, numerous studies have highlighted the beneficial effects of traditional fermented foods on overall health. These foods have been shown to reduce the risk of cardiovascular disease, improve blood sugar and lipid profiles, alleviate constipation and diabetes symptoms, boost the immune system, and exhibit anti-cancer properties [11,12,13]. During fermentation, nutrients become more bioavailable, and probiotics play a crucial role in this process, further promoting health [14]. One such traditional fermented food is Chinese chives, which contain a dominant strain of *Lactiplantibacillus plantarum* (*L. plantarum*) [15]. *L. plantarum* has been found to offer potential functions, including reducing serum cholesterol and providing anti-oxidation effects that help lower the risk of cardiovascular disease [16,17]. Additionally, *L. plantarum* strains have been demonstrated to regulate the gut microbiota by producing bacteriocins that target various harmful bacteria, including *Staphylococcus aureus*, *Listeria monocytogenes* (Gram-positive), *Escherichia coli*, *Salmonella* spp., and *Shigella* spp. [18]. *L. plantarum* has also been shown to influence conditions such as obesity, ulcerative colitis, depression, and cardiovascular disease by regulating gut microbes [19,20,21,22]. These findings suggest that *L. plantarum* has the potential to ameliorate disease by modulating the gut microbiota.

However, it is still unknown whether *L. plantarum* P470 isolated from fermented Chinese chives could modulate gut microbiota and metabolic status in feces obtained from CHD patients. In this study, we investigated the effects of *L. plantarum* P470 on antioxidant and anti-inflammatory activities, as well as its impact on gut microbiota and metabolic profiles in fecal samples from CHD patients, using an in vitro anaerobic fermentation model. The objective is to explore how beneficial bacteria from traditional fermented foods can modulate the fecal microbiota in individuals with cardiovascular disease.

## 2. Materials and Methods

### 2.1. Materials

The *L. plantarum* P470 strain, derived from fermented Chinese chives in Xinjiang, was obtained from the Institute of Microbiology, Guangdong Academy of Sciences (Guangzhou, China). The strain was cultured in De Man, Rogosa, and Sharpe (MRS) broth at 37 °C for 18 h. Escherichia coli O111:B4 lipopolysaccharide (LPS) and standards for short-chain fatty acids (SCFAs) were purchased from Sigma-Aldrich Chemical Co., Ltd. (St. Louis, MO, USA). The RAW264.7 mouse monocyte macrophage cell line was also sourced from the Institute of Microbiology, Guangdong Academy of Sciences (Guangzhou, China). Dulbecco’s Modified Eagle Medium (DMEM), streptomycin, penicillin, trypsin-EDTA, and fetal calf serum (FCS) were acquired from Gibco/Invitrogen (Carlsbad, CA, USA).

### 2.2. In Vitro Colonic Fermentation Models

Stool samples were obtained from both a healthy population (HP) and patients with CHD who had no history of gastrointestinal disorders and had not consumed probiotics or antibiotics in the preceding four weeks. The fecal samples from CHD patients were obtained from the First Affiliated Hospital of Guangdong Pharmaceutical University, and informed consent was obtained from all participating volunteers. The fermentation of *L. plantarum* P470 in vitro was conducted following the procedures described by Chen et al. [23], we made several detailed adjustments. The base nutrient medium was prepared by dissolving 0.20 g of peptone, 0.40 g of yeast extract, 0.01 g of sodium chloride, 4 mg of K_2_HPO_4_, 4.00 mg of KH_2_PO_4_, 1 mg of MgSO_4_, 1 mg of CaCl_2_, 20 mg of chlorhematin, 46 mg of cysteine hydrochloride, 50 mg of bile salts, 0.10 mg of resazurin, 0.20 mL of Tween 80, and 1.00 μL of vitamin K1 in 100 mL of distilled water. The pH of the nutrient medium was adjusted to 7 with 0.50 M NaHCO_3_, and the medium was sterilized by autoclaving. Fecal samples were collected from four healthy volunteers and four patients with CHD (one female, three males, 60 to 75 years old). Fecal samples from HP and CHD patients were combined in equal volumes with sterilized saline at a 1:10 ratio (*w*:*v*) and centrifuged at 500× *g* for 5 min at 4 °C. Then, 1.00 mL of the supernatant from each group was added to 4 mL of autoclaved basal nutrient medium to form the HP-48 and CHD-48 groups. Next, 0.10 mL of *L. plantarum* P470 (10^9^ CFU) was added to 4 mL of the same medium with 1.0 mL of fecal supernatant, creating the HP-48-LP and CHD-48-LP groups. The fermentation groups, including HP-48, CHD-48, HP-48-LP, and CHD-48-LP groups, were incubated in an Anaero Pack System (Mitsubishi Gas Chemical Co., Inc., Tokyo, Japan) at 37 °C for 48 h. After centrifugation at 7000× *g* for 5 min, the supernatant and pellet were collected for further experiments.

### 2.3. Determination of Antioxidant Activity before and after Fermentation

The antioxidant activity of the fermentation supernatants from the HP-48, CHD-48, HP-48-LP, and CHD-48-LP groups was evaluated using both the 1,1-diphenyl-2-picrylhydrazyl (DPPH) free radical scavenging assay and the hydroxyl radical scavenging assay. The DPPH free radical scavenging assay was conducted with modifications to the method described by [24]. In a 96-well plate, 100 μL of each sample was combined with 100 μL of 0.20 mM DPPH solution (Yuanye Biology, Shanghai, China). The mixture was incubated in the dark at room temperature for 30 min. After incubation, the absorbance at 517 nm (A517) was measured and recorded as Ai. The DPPH scavenging effect was determined using the following formula:

(1) $$\mathrm{Scavenging}\;\mathrm{effect}\;\left(\%\right)=\left[\frac{\mathrm{Ac}-\mathrm{Aj}}{\mathrm{Ac}}\right]\times 100$$

where Aj is A517 of DPPH with the basal nutrient medium sample, and Ac is A517 with distilled water and DPPH. 

The hydroxyl radical scavenging assay was conducted following the methodology outlined in the referenced publication [25]. Briefly, a mixture was prepared comprising 30 μL of 0.75 mM phenanthrene solution, 60 μL of 0.20 M phosphate buffer solution (PBS) with a pH of 7.40, 30 μL of 0.75 mM FeSO_4_, 30 μL of the sample solution, and 30 μL of 0.01% (*v*/*v*) H_2_O_2_. The samples were then incubated in a water bath at 37 °C for 60 min, after which the absorbance was measured at 536 nm.

(2) $$\mathrm{Hydroxyl}\;\mathrm{radical}\;\mathrm{scavenging}\;\mathrm{activity}\;\left(\%\right)=[\frac{\left(\mathrm{Aa}-A0\right)}{\left(\mathrm{Ab}-A0\right)}]\times 100$$

where Aa is 30 μL of sample solution and 30 μL of 0.01% (*v*/*v*) H_2_O_2_; Ab is 30 μL distilled water and 30 μL sample; A0 is 30 μL distilled water and 30 μL of 0.01% (*v*/*v*) H_2_O_2_.

### 2.4. In Vitro Assessment of Anti-Inflammatory Effects

#### 2.4.1. Measurement of Cell Counting Kit-8 (CCK8) in RAW264.7 Cells

RAW264.7 is the most commonly used inflammatory cell model. It is difficult to establish inflammatory models on colon cell lines as there is little literature, so RAW264.7 was selected as the object of inflammation research. The RAW264.7 cells were cultured in DMEM with 10% FCS, 100 μg/mL of penicillin, and 100 μg/mL of streptomycin at 37 °C with 5% CO_2_. Initially, 10^4^ cells in 100 μL of suspension were plated into each well of a 96-well plate and incubated for 24 h. The following day, the supernatant was discarded, and the cells were rinsed twice with PBS. Then, 90 μL of DMEM culture medium and 10 μL of sterile fermentation broth at various concentrations were added to the wells. After 24 h incubation, 10 μL of Cell Counting Kit-8 (CCK-8) solution was introduced to each well. The plate was incubated for a further 4 h, after which absorbance at 450 nm was measured. The cell survival rate was determined using the formula:

(3) $$\mathrm{Survival}\;\mathrm{rate}\;\left(\%\right)=[\frac{\left(\mathrm{Ba}-B0\right)}{\left(\mathrm{Bb}-B0\right)}]\;\times \;100$$

where Ba is 90 μL of DMEM with varying concentrations of the sample solution and 10 μL of CCK8; Bb is 90 μL of DMEM with 10 μL of CCK8; B0 is 90 μL of DMEM with 10 μL of CCK8 but without cells.

#### 2.4.2. In Vitro Evaluation of Anti-Inflammatory Effects

The 5 × 10^5^ RAW264.7 cells were seeded into 12-well culture plates and subsequently incubated at 37 °C with 5% CO_2_. On the second day, the old medium was removed after 24 h of incubation, and fresh DMEM minus any concentration causing cell damage (survival rate > 95%) was added to each well. Following a 1 h incubation period, LPS (at a final concentration of 1 μg/mL) was added to each group, excluding the blank control group. The cells were incubated for an additional 24 h. Subsequently, RNA was extracted using the RNA Easy Fast Animal Tissue/Cell Total RNA Extraction Kit (Magen Biotech Co., Ltd., Guangzhou, China). The expression levels of inflammatory factors (TNF-α, IL-6, and IL-10) were then measured by fluorescent quantitative PCR (qPCR) using the LightCycler^®^ 480 SYBR Green I Master system (Roche, Basel, Switzerland). The primer sequences for the relevant genes used in the experiment are listed in Table 1. The relative mRNA levels of TNF-α, IL-6, and IL-10 were determined using the 2^−ΔΔCt^ method.

### 2.5. DNA Extraction and Gut Microbiota Analysis

Centrifuge the fermentation solution at 10,000× *g* for 5 min to collect the pellet. Extract bacterial DNA from the pellet using a commercial kit (QiAamp DNA Stool Mini Kit, Hilden, Germany). After the DNA samples pass amplification testing, the PCR products are mixed and purified. Subsequently, end repair, A-tailing, adapter ligation, and further purification are performed to complete the library preparation. The hypervariable regions V3-V4 (341F: CCTAYGGGRBGCASCAG, 806R: GGACTACNNGGGTATCTAAT) of the bacterial 16S rRNA gene were sequenced by Novogene Co., Ltd. (Beijing, China) using the Illumina NovaSeq PE250 (California, USA) strategy for double-ended sequencing. The open-source platform QIIME 2 (202202) was used to process and filter the raw data to obtain clean data. The DADA2 method was then employed to de-noise the clean data and filter sequences with an abundance of less than 5, resulting in the final ASV table. Subsequently, species annotations and phylogenetic trees were generated from the ASV table, followed by alpha diversity analysis and diversity analysis. The observed_otus index was used to plot sparse curves, Chao1 and Shannon indexes were used for alpha diversity analysis, and the PCoA plot (unweighted UniFrac distance) was used to depict beta diversity indices.

### 2.6. Gas Chromatography for SCFA Analysis

First, feces samples were treated with 0.001% sulfuric acid and centrifuged for 13,000× *g* for 25 min, and the supernatant was collected. Then, the concentration of SCFAs in the supernatant was measured using an Agilent 7890A gas chromatograph (Agilent Technologies, Santa Clara, CA, USA), with chromatography performed using a Thermo TG-624S1MS (30 m × 320 µm i.d., 1.8 µm). The experiment utilized specific parameters, including a pressure of 7.5649 pounds per square inch (psi), a desolvation gas flow rate of 10 milliliters per minute (mL/min), a cone gas flow rate 3 mL/min, and a cylinder temperature ranging from 60 to 180 °C. Additionally, an ion flame detector was used with an inlet temperature set to 250 °C. Nitrogen was employed as the carrier gas. Short-chain fatty acids in the samples were determined by an external standard method. Retention times and peak areas of the samples were determined through the analysis of a standard mixture [26].

### 2.7. UPLC-MS/MS for Untargeted Metabolomics

Firstly, 100 μL of fermentation supernatant was centrifuged at 13,000× *g* for 25 min. The resulting pellet was dissolved in 250 μL of a pre-cooled mixture of methanol, acetonitrile, and water (4:2:2; *v*:*v*:*v*). Subsequently, the mixture was subjected to ultrasonication in a water bath for 10 min, followed by refrigeration at −20 °C for 1 h and centrifugation at 12,000× *g* at 4 °C for 10 min. The supernatant was collected and evaporated to dryness at room temperature using a vacuum drying oven. The dried sample was reconstituted in 200 μL of chilled acetonitrile and water (1:1, *v*:*v*) and then centrifuged at 12,000 rpm at 4 °C for 15 min. Both the supernatant and quality control (QC) samples were analyzed with the same loading volume [27]. The raw data from the UPLC UltiMate3000 system were processed using Thermo Compound Discoverer™ 3.1 software. Metabolite structures were identified by consulting the HMDB (http://www.hmdb.ca/), KEGG (http://www.kegg.jp/), and ChemSpider (http://www.chemspider.com/) databases, accessed on 1 February 2023. The raw data were then normalized using summation normalization.

### 2.8. Statistic Analysis

The data are expressed as mean ± standard deviation (SD). Statistical analyses were performed using SPSS 25 software (IBM), with either a *t*-test or Mann–Whitney test applied based on the data distribution. A significance level of (*p* < 0.05) was considered statistically significant. Using the RStudio to create plots related to gut microbiota. Additional metabolite analyses were conducted using GraphPad Prism 8 software (GraphPad Software, La Jolla, CA, USA) and MetaboAnalyst (https://www.metaboanalyst.ca/), accessed on 20 February 2023. Compounds with (*p*)-values less than 0.05 and fold changes greater than 2 or less than 0.5 were classified as differential metabolites.

## 3. Results

### 3.1. L. plantarum P470 Exerts an Antioxidant Activity in Feces from CHD Patients

**Figure 1:**
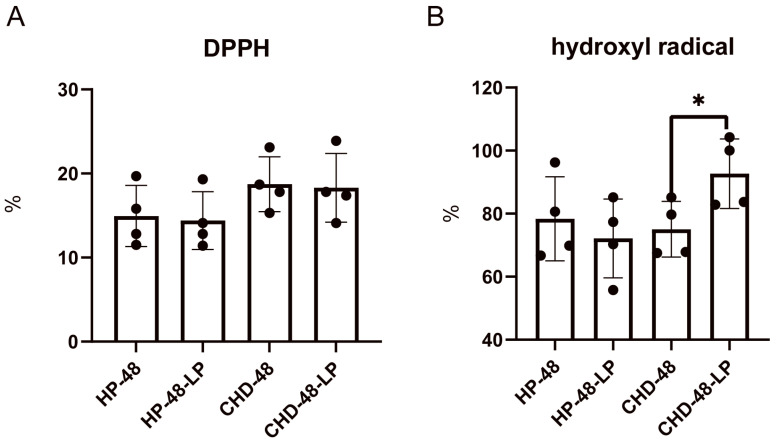
The effects of *L. plantarum* P470 on antioxidant ability. (**A**), 1,1-Diphenyl-2-picrylhydrazyl radical (DPPH) free radical scavenging assay, (**B**), hydroxyl radical scavenging assay. Significant differences between the CHD-48-LP group and the CHD-48 group, as well as between the HP-48-LP group and the HP-48 group, were assessed using either a *t*-test or Mann–Whitney test (* *p* < 0.05). The results are presented as means from the data (*n* = 4).

To investigate whether the fermentation of human feces by *L. plantarum* P470 can enhance the antioxidant activity of the supernatant, we evaluated the fermentation supernatant’s DPPH and hydroxyl radical antioxidant activities. As shown in Figure 1, after 48 h of fermentation, no significant difference was observed in DPPH antioxidant activity among the groups. However, the level of hydroxyl radical antioxidant activity was significantly increased after *L. plantarum* P470 fermentation of feces from patients with CHD (CHD-48-LP group, 92.68 ± 11.04%) compared to the CHD-48 group (78.06 ± 8.80%). No significant difference was observed in fermentations using feces from healthy individuals. This discrepancy is likely due to differences in the fecal environment between healthy and diseased populations. Based on these results, we conclude that *L. plantarum* P470 has the ability to enhance the hydroxyl radical antioxidant capacity in the feces of CHD patients.

### 3.2. L. plantarum P470 Seems to Exert Some Anti-Inflammatory Activity in Feces of CHD Patients

**Figure 2:**
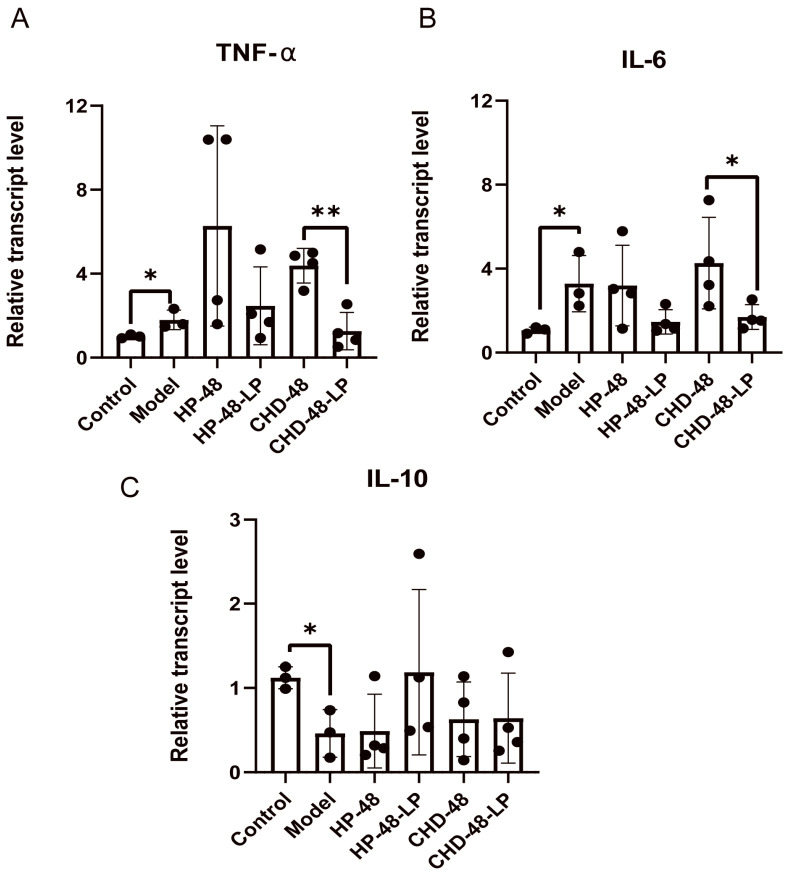
The effect of fermentation solution on the expression of TNF- α (**A**), IL-6 (**B**), and IL-10 (**C**) in RAW264.7 macrophage cells. Significant differences between the CHD-48-LP group and the CHD-48 group, or the HP-48-LP group and the HP-48 group, were analyzed using a *t*-test or Mann–Whitney test (* *p* < 0.05, ** *p* < 0.01). Results are reported as means of the data (*n* = 4).

The exact role of *L. plantarum* P470 in mediating the anti-inflammatory effects observed in human feces remains uncertain. Hence, we conducted experiments using RAW264.7 cells to explore whether *L. plantarum* P470 has the potential to inhibit the inflammatory responses induced by LPS. As depicted in Figure 2, exposure to LPS resulted in a significant elevation in TNF-α and IL-6 expression, along with a decrease in IL-10 expression compared to the control group. However, in the CHD-48-LP group, there was a noteworthy decrease in TNF-α and IL-6 expression (1.27 ± 0.89, 1.69 ± 0.51, respectively) compared to the CHD-48 group (*p* < 0.05, 4.38 ± 0.83, 4.27 ± 1.89, respectively). In addition, the expression levels of TNF-α and IL-6 were also found to be lower in the HP-48-LP group compared to the HP-48 group. Although the expression level of IL-10 in HP-48-LP was increased, there was no significant difference compared to the HP-48 group. While the levels of TNF-α and IL-6 in the HP-48-LP group (2.46 ± 1.86, 1.46 ± 0.50, respectively) were decreased compared to the HP-48 group (6.27 ± 4.77, 3.19 ± 1.66, respectively), our findings indicate that *L. plantarum* P470 may enhance the anti-inflammatory capacity of human feces, particularly in individuals with CHD.

### 3.3. L. plantarum P470 Exerts Restricted Efficacy in Feces from CHD Patients In Vitro

**Figure 3:**
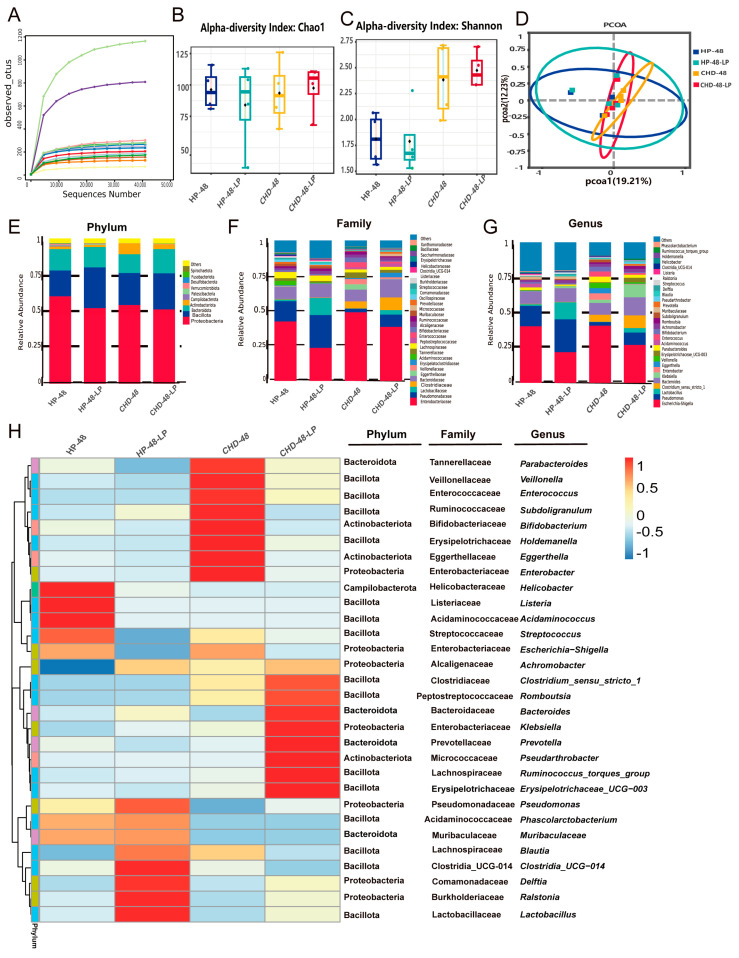
The effects of *L. plantarum* P470 on gut microbial structure and relative abundance. (**A**) observed_otus rarefaction curve with sample; (**B**) Chao1 alpha-diversity index; (**C**) Shannon alpha-diversity index; (**D**) beta-diversity of PCoA; (**E**) the relative abundance at phylum level; (**F**) the relative abundance at family level; (**G**) the relative abundance at genus level; (**H**) correlation heatmap at genus level, between the CHD-48-LP and CHD-48 groups, and between the HP-48-LP and HP-48 groups, were assessed using a *t*-test or Mann–Whitney test. Results are presented as means of the data (*n* = 4).

The impact of *L. plantarum* P470 on gut microbiota was examined using high-throughput sequencing of 16S rRNA genes. As the number of sequences increased, the observed OTUs reached a plateau (Figure 3A), indicating that the data were sufficient for further analysis. The effects of *L. plantarum* P470 on microbial composition and diversity were assessed through analyses of alpha-diversity (Chao1, Shannon) and beta-diversity (PCA) (Figure 3B–D). The analysis revealed no significant differences between CHD-48 and CHD-48-LP or between HP-48 and HP-48-LP in these indices. Our findings suggest that *L. plantarum* P470 has limited efficacy in altering the composition and diversity of gut microbiota.

However, the taxonomic profile data revealed distinct variations in the gut microbiota structure among the groups. At the phylum level, Proteobacteria, Bacillota, Bacteroidota, and Actinobacteriota, were the dominant gates among the four groups after 48 h of fermentation. Although there were no notable differences between the groups, the ratio of Bacillota to Bacteroidota decreased from 2.35 to 2.00 in the HP group and from 1.73 to 1.49 in the CHD group after treatment with *L. plantarum* P470 in vitro. The results suggested that *L. plantarum* P470 may enhance the abundance of Bacteroidota (Figure 3E, Table S1). At the family level, the gut microbiota in patients with CHD showed a higher proportion of Enterobacteriaceae, Clostridiaceae, Eggerthellaceae, Veillonellaceae, Tannerellaceae, Erysipelotrichaceae, and a lower relative abundance of Bacteroidaceae, Lachnospiraceae, Prevotellaceae, Lactobacillaceae compared to the HP groups (Figure 3F, Table S2). Additionally, *L. plantarum* was observed to enhance the gut microbiota in patients with CHD by reducing the relative prevalence of Enterobacteriaceae, Eggerthellaceae, Veillonellaceae, Tannerellaceae, and Erysipelotrichaceae, while augmenting the presence of Bacteroidaceae, Prevotellaceae, Lactobacillaceae. The results indicated the *L. plantarum* P470 could alter the abundance of some of the family, notably decreasing levels of Eggerthellaceae and Erysipelotrichaceae, and increasing the levels of Prevotellaceae, Lactobacillaceae in the CHD-48-LP group compared to CHD-48 group. In terms of genus level, the relative abundance of *Escherichia-Shigella*, *Clostridium_sensu_stricto_1*, *Enterobacter*, *Eggerthella*, *Veillonella*, *Erysipelotrichaceae_UCG-003*, *Enterococcus*, *Subdoligranulum*, *Holdemanella* was increased, while the abundance of *Lactobacillus*, *Bacteroides*, *Prevotella*, *Ralstonia* was decreased in CHD-48 group, compare to HP-48 group. However, *L. plantarum* P470 significantly reduced the abundance of *Enterobacter*, *Eggerthella*, *Subdoligranulum*, *Holdemanella*, while increasing the proportion of *Lactobacillus*, *Ralstonia* in the CHD-48-LP group compared with CHD-48 group (Figure 3G,H, Table S3, *p* < 0.05). Notably, *Escherichia-Shigella*, *Bacteroides*, and *Veillonella* were found to be the most significantly impacted bacteria in the gut microbiota of patients with CHD. Specifically, supplementation with *L. plantarum* P470 led to a rise in the relative presence of *Bacteroides*, increasing from 8.71 ± 8.88% (CHD-48 group) to 13.32 ± 15.81% (CHD-48-LP group). Additionally, it resulted in a reduction in the relative abundance of *Escherichia-Shigella* from 42.04 ± 13.93% (CHD-48 group) to 27.91 ± 12.53% (CHD-48-LP group), as well as a decrease in the relative abundance of *Veillonella* from 3.8 ± 7.52% (CHD-48 group) to 1.11 ± 2.21% (CHD-48-LP group), although these differences did not reach statistical significance. Furthermore, the levels of *Enterobacter*, *Veillonella*, *Eggerthella*, and *Streptococcus* were significantly decreased, and the relative abundance of *Lactobacillus* was significantly elevated in HP-48-LP compared to HP-48 (Figure 3G, Table S3, *p* < 0.05), primarily modulating the abundance of genera such as *Escherichia-Shigella*, *Enterobacter*, *Veillonella*, *Eggerthella*, *Bacteroides*, and *Lactobacillus*, thereby improving the gut microbiota in the feces of CHD patients.

**Figure 4:**
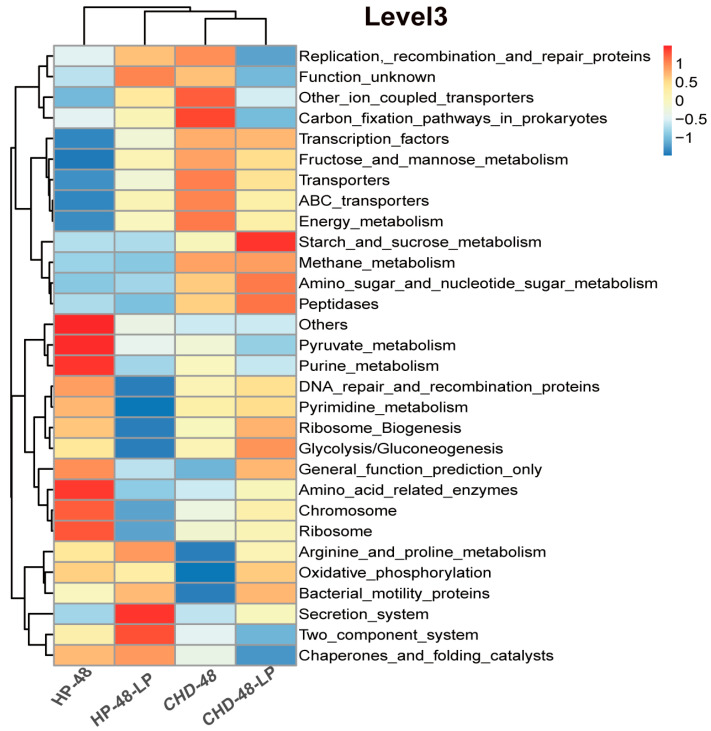
PICRUSt function prediction based on Level 3.

The researchers utilized the PICRUSt algorithm to predict the functional contributions of genomes based on 16S rRNA sequence data. The top 30 KEGG orthologs (KOs) at level 3, as determined by the functional annotation of differentially expressed genes across the four study groups, are presented in Figure 4. Compared to the HP-48 group, the CHD-48 group exhibited lower levels of arginine and proline metabolism, bacterial motility proteins, oxidative phosphorylation, and amino acid-related enzymes. In contrast, the CHD-48 group showed higher levels of energy metabolism, fructose and mannose metabolism, and carbon fixation pathways in prokaryotes. Importantly, *L. plantarum* P470 was found to enhance the pathways related to arginine and proline metabolism, glycolysis/gluconeogenesis, bacterial motility proteins, and the secretion system in both the CHD and HP groups. Additionally, *L. plantarum* P470 increased the levels of amino sugar and nucleotide sugar metabolism, Starch and sucrose metabolism, peptidases, and amino acid-related enzymes in the CHD-48-LP group compared to the CHD-48 group. Thus, the main effect of *L. plantarum* P470 appears to be the modulation of gut microbiota in the feces of CHD patients, leading to alterations in microbiota function, particularly in amino acid-related enzymes, peptidases, arginine and proline metabolism, and glycolysis/gluconeogenesis.

### 3.4. L. plantarum P470 Enhances Fecal SCFAs Content of CHD Patients In Vitro

**Figure 5:**
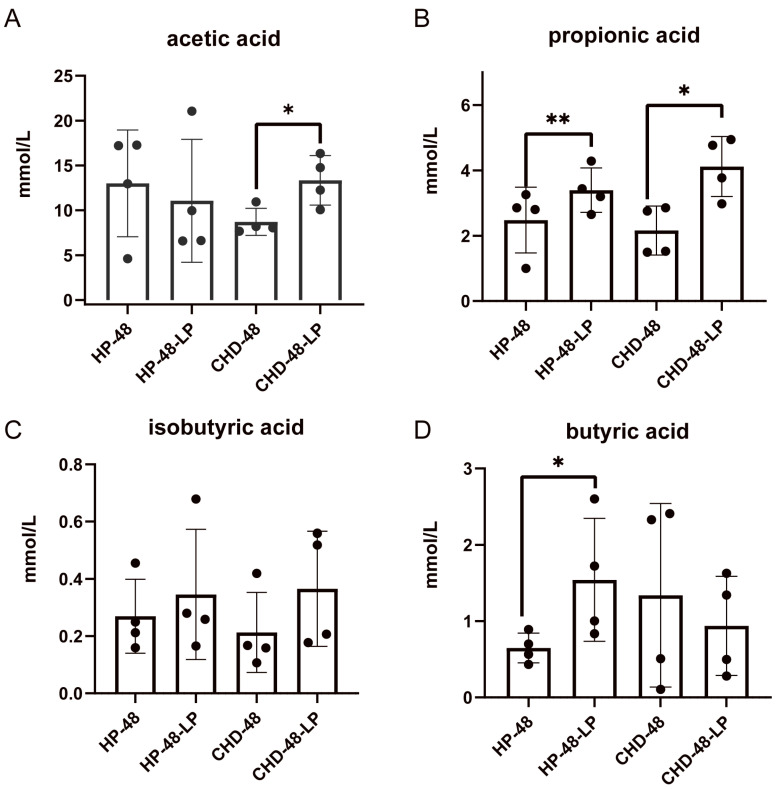
The contents of major short-chain fatty acids during 48 h of fermentation with L. plantarum P470: (**A**) acetic acid; (**B**) propionic acid; (**C**) isobutyric acid; (**D**) butyric acid. Significant differences between the CHD-48-LP and CHD-48 groups or between the HP-48-LP and HP-48 groups (* *p* < 0.05, ** *p* < 0.01) were analyzed using the *t*-test or Mann–Whitney test. Results are presented as means (*n* = 4).

Abundant evidence underscores the crucial role of short-chain fatty acids (SCFAs) in maintaining gut and metabolic health. This explains why modifications in gut microbiota can affect disease pathophysiology [28]. To further investigate the potential health-promoting functions of *L. plantarum* P470, we measured SCFAs using gas chromatography (GC). As shown in Figure 5, we quantified the levels of acetic acid, propionic acid, isobutyric acid, and butyric acid, which are primary fermentation metabolites. Our results indicated that *L. plantarum* P470 significantly increased the levels of acetic acid and propionic acid in the CHD-48-LP group compared to the CHD-48 group (13.35 ± 2.76 mmol/L vs. 8.71 ± 1.50 mmol/L, 4.12 ± 0.92 mmol/L vs. 2.15 ± 0.74 mmol/L, respectively; *p* < 0.05). Additionally, *L. plantarum* P470 significantly elevated the levels of propionic acid and butyric acid in the HP-48-LP group compared to the HP-48 group (3.39 ± 0.68 mmol/L vs. 2.47 ± 1.00 mmol/L, 1.54 ± 0.80 mmol/L vs. 0.64 ± 0.19 mmol/L, respectively; *p* < 0.05). Although no significant difference in isobutyric acid levels was observed among the groups, an upward trend in fermentation was noted with *L. plantarum* P470 in both the CHD-48-LP and HP-48-LP groups. Therefore, our findings suggest that *L. plantarum* P470 can enhance the production of SCFAs in human feces, potentially contributing to its health-promoting benefits.

### 3.5. L. plantarum P470 Regulated Metabolism in Feces of CHD Patients In Vitro

**Figure 6:**
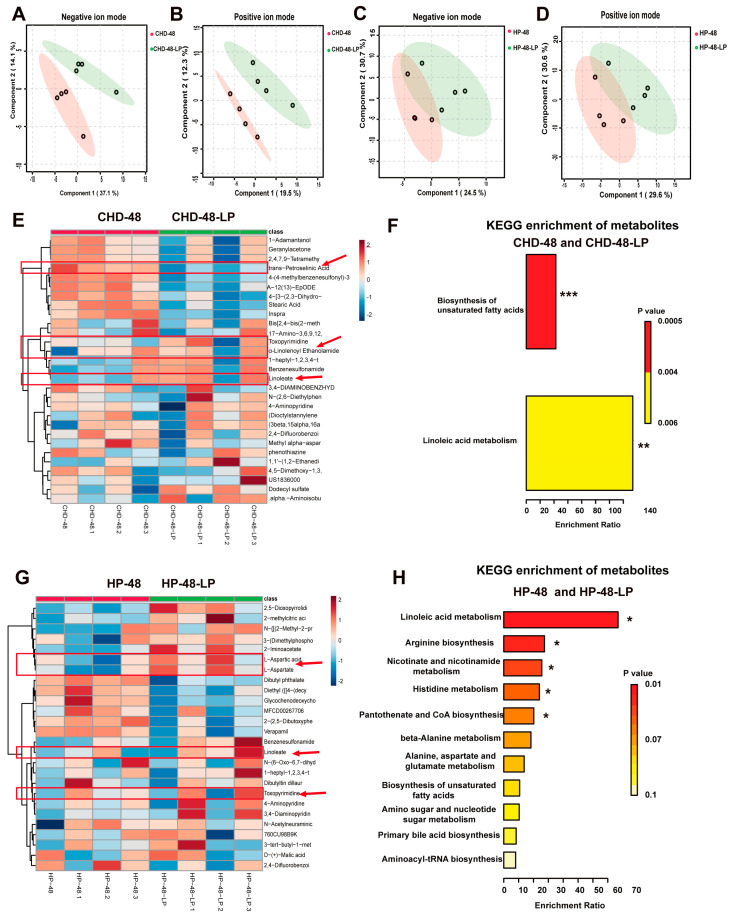
The impact of *L. plantarum* P470 on metabolites in fermentation supernatants, as assessed by untargeted metabolomic analysis using UPLC-MS/MS. (**A**) Partial least squares discriminant analysis (PLS-DA) of negative models comparing the CHD-48-LP group to the CHD-48 group; (**B**) PLS-DA of positive models comparing the CHD-48-LP group to the CHD-48 group; (**C**) PLS-DA of negative models comparing the HP-48-LP group to the HP-48 group; (**D**) PLS-DA of positive models comparing the HP-48-LP group to the HP-48 group; (**E**) correlation heat map of differential metabolites for the CHD-48-LP group versus the CHD-48 group; (**F**) KEGG enrichment analysis of differential metabolites for the CHD-48-LP group versus the CHD-48 group (** *p* < 0.01, *** *p* < 0.001); (**G**) correlation heat map of differential metabolites for the HP-48-LP group versus the HP-48 group; (**H**) KEGG enrichment analysis of differential metabolites for the HP-48-LP group versus the HP-48 group (* *p* < 0.05).

To assess the influence of *L. plantarum* P470 treatment on the metabolite profile, we conducted an untargeted metabolomic analysis on the fermentation supernatant collected from both healthy individuals and those with CHD. We utilized partial least squares—discriminant analysis (PLS-DA) to visualize the distribution of positive and negative ion scanning data across the two groups. The PLS-DA plot exhibited a clear clustering of metabolites from each group, demonstrating distinct separation (Figure 6A–D). Differential metabolites were defined as those with *p* < 0.05 and fold change of >2 or <0.5. We conducted cluster analysis on the distinct metabolites found in the fermentation supernatant obtained from both the healthy and CHD groups. Our results showed that the levels of α-linolenoyl ethanolamide, linoleate, and toxopyrimidine were higher, while the content of trans-petroselinic acid, A-12(13)-EpODE, and stearic acid were lower in the CHD-48-LP group compared to the CHD-48 group (Figure 6E, Table 2). Moreover, differential metabolite enrichment pathways were analyzed using the Kyoto Encylopaedia of Genes and Genomes (KEGG). These differential metabolites were significantly enriched in the pathways for Biosynthesis of unsaturated fatty acids and linoleic acid metabolism (Figure 6F). In the HP-48-LP group, the levels of L-aspartic acid, L-aspartate, linoleate, and toxopyrimidine were significantly increased, while the level of glycochenodeoxycholic acid was lower when compared to the HP-48 group (Figure 6F, Table 2). These differential metabolites were mainly enriched in the Linoleic acid metabolism, arginine biosynthesis, nicotinate and nicotinamide metabolism, histidine metabolism, pantothenate and CoA biosynthesis, beta-alanine metabolism, alanine, aspartate and glutamate metabolism, and biosynthesis of unsaturated fatty acids pathways. Notably, the content of linoleate and toxopyrimidine was significantly increased following treatment with *L. plantarum* P470 in fermentation supernatant when compared to the HP-48 and CHD-48 groups. Our findings suggest that *L. plantarum* P470 mainly regulates the biosynthesis of unsaturated fatty acids and linoleic acid metabolism in fermentation metabolites in vitro.

## 4. Discussion

Cardiovascular disease (CVD) is a leading global cause of mortality, characterized by the rising prevalence of CHD and atherosclerosis [29]. Research has shown that the structure and function of the gut microbiota are related to CHD [30]. Probiotics and prebiotics have gained attention in recent years due to their potential for disease prevention by modulating the gut microbiota [31]. Fermented Chinese chives, a traditional food in China, have been found to offer potential benefits for human health [32], with the dominant strain *L. plantarum* possibly contributing to these effects. In this study, *L. plantarum* P470, isolated from fermented Chinese chives, was used to investigate its effects on anti-oxidation, anti-inflammatory responses, gut microbiota, and metabolic status in the feces of CHD patients using an anaerobic fermentation model in vitro.

The antioxidant activity against hydroxyl free radicals in the fermentation broth may be related to the fermentation metabolites [33], which further supports the notion that *L. plantarum* P470 ferments human feces to alter the metabolic state of the fecal microenvironment, often with beneficial effects. The results of this study demonstrated that *L. plantarum* P470 improved the scavenging ability of hydroxyl radicals in the feces of CHD patients but showed no significant difference in the feces of healthy individuals. This suggests that different metabolic states exhibit varying antioxidant activities, warranting further sample verification. TNF-α and IL-6 are key inflammatory factors in cardiovascular diseases [34]. During inflammation, macrophages are recruited to the inflamed area and engulf modified LDL particles, leading to the formation of lipid-laden foam cells. These foam cells exacerbate local inflammation by secreting pro-inflammatory mediators, including cytokines like TNF-α, and trigger the production of secondary signaling cytokines such as IL-6 [3]. The results of the anti-inflammatory experiment in the fermentation supernatant indicated that *L. plantarum* P470 significantly reduced the levels of TNF-α and IL-6 in the stool of CHD patients, while these levels showed a downward trend in healthy individuals. This finding suggests that *L. plantarum* P470 improves the anti-inflammatory state during fecal fermentation, particularly in CHD feces.

After 48 h of fermentation, *L. plantarum* P470 was found to modulate the gut microbiota in the feces of patients with CHD. At the phylum level, the ratio of Bacillota to Bacteroidota (F/B) was reduced following treatment with *L. plantarum* P470 in CHD gut microbiota in vitro. Stool samples obtained from individuals with atherosclerosis demonstrated elevated levels of the Bacillota phylum and reduced abundance of the Bacteroidetes phylum compared to samples from healthy controls [35]. Emoto et al. proposed that a decreased occurrence of the Bacteroidota phylum and an elevated F/B in the gastrointestinal tract might be associated with the development of coronary artery disease [36]. Bacteroidota were found to be enriched in various carbohydrate metabolism pathways, while Bacillota bins showed a higher enrichment in transport systems [37]. The study also found that *L. plantarum* P470 could increase the bacterial function of several metabolic pathways, including Arginine_and proline_metabolism, glycolysis/gluconeogenesis, bacterial_motility proteins, amino_sugar and_nucleotide sugar metabolism, Starch and sucrose metabolism using PICRUSt algorithm, compared with CHD-48 group. This increase in bacterial function may be related to the increased abundance of Bacteroidetes in CHD patient feces.

Imbalanced ratios of Bacillota to Bacteroidetes are a major cause of intestinal microbial imbalance, and adjusting this ratio has received extensive attention. Moreover, Veillonellaceae, a family within the Bacillota phylum, has been observed to have a favorable association with metabolic disorders triggered by high-fat diets [38], while Eggerthella lenta (Acfinobacteria phylum) has been observed to stimulate intestinal inflammation by stimulating the expression of Rorc and genes associated with Th17 [39,40,41]. Additionally, in functional studies, *Eggerthella lenta* has been implicated in the conversion of L-carnitine to TMA [42]. Our findings revealed the presence of *Veillonella* and *Eggerthella* were decreased following treatment with *L. plantarum* P470 in feces of CHD gut microbiota in vitro. 

Proteobacteria is a phylum that contains more pathogenic microorganisms, including *Escherichia/Shigella*, *Helicobacter*, and *Campylobacter*, which are found in higher abundance in CHD patients with atherosclerosis [43]. Previous research has shown that Proteobacteria was positively correlated with adipose inflammatory factors [44], suggesting a potential link between this phylum and CVD risk factors. TMA-producing bacteria, such as *Escherichia-Shigella*, have been found to aggravate the progression of atherosclerosis [45], further emphasizing the importance of regulating microbial composition in relation to CHD. Additionally, *Helicobacter pylori* infection has been suggested as a risk factor for CHD, especially in individuals under 60 years old and without cardiovascular risk factors [46]. In this study, the abundance of Proteobacteria, specifically *Escherichia-Shigella*, *Enterobacter*, and *Helicobacter*, was found to be higher in the CHD-48 group but decreased following the addition of *L. plantarum* P470. These findings suggest that *L. plantarum* P470 isolated from fermented Chinese Chives may have the potential to improve fecal physiological status in patients with CHD by regulating the microbiota and reducing the abundance of pathogenic microorganisms such as *Escherichia-Shigella*, *Enterobacter*, and *Helicobacter*.

Short-chain fatty acids (SCFAs), including acetate, propionate, and butyrate, are essential for maintaining intestinal homeostasis. During atherosclerosis development, SCFAs are recognized as beneficial gut microbial products with anti-atherosclerotic effects, such as inhibiting systemic inflammation, promoting endothelial cell function, and maintaining intestinal barrier integrity [47,48]. Previous research has shown that under lipopolysaccharide (LPS) stimulation, SCFAs at concentrations ranging from 0.20 to 20 mmol/L can decrease the production of TNF-α and monocyte chemotactic protein-1 (MCP-1) [49]. Furthermore, butyrate treatment of Ea.hy926 cells has been found to reduce ox-LDL uptake, CD36, VCAM-1, TNF-α, and interleukin-1β/6 production while increasing IL-10 production, suggesting its potential as a treatment for atherosclerosis [50]. In this study, it was observed that *L. plantarum* P470 significantly elevated the levels of acetic acid and propionic acid in the feces of CHD patients and increased propionic acid and butyric acid levels in the group with *Helicobacter pylori* infection (HP) (*p* < 0.05). These findings suggest that *L. plantarum* P470 holds promise as a potential strategy for enhancing host health and as a treatment approach for CHD patients. Additionally, the fermentation solution administered to the CHD-48-LP group resulted in significant reductions in TNF-α and IL-6 levels (*p* < 0.05) while increasing IL-10 content in the HP-48-LP group, indicating that *L. plantarum* P470 may regulate the intestinal microbiota to produce SCFAs, thereby playing an anti-inflammatory role. 

Linoleic acid (LA, ω-6 18:2) is highly susceptible to oxidation in the presence of lipoxygenase, cytochrome P450, metal ions, and free radicals. LA can be converted to the epoxide epoxyeicosatetraenoic acid (EPODE), one of the isoforms known to cause pulmonary edema in pathophysiological studies [51,52]. Several studies employing mixed supplementation strategies with conjugated linoleic acid (CLA) have demonstrated a reduction in the size of atherosclerotic lesions in the aorta, accompanied by decreased accumulation of macrophages and a decrease in the expression of pro-inflammatory genes [53,54]. Both 9,11-CLA and 10,12-CLA have been observed to inhibit the expression of crucial monocyte adhesion molecules found on endothelial cells, namely vascular cell adhesion molecule-1 (VCAM-1) and intercellular adhesion molecule-1 (ICAM-1) [55]. In this work, we found that *L. plantarum* P470 can significantly increase the levels of LA in the CHD patient group and HP group. In this study, we found that *L. plantarum* P470 significantly increased the levels of LA in both the CHD patient group and the HP group. Additionally, *L. plantarum* P470 significantly reduced the level of A-12(13)-EpODE and increased hydroxyl radical scavenging ability in the CHD patient group, suggesting an improvement in antioxidant capacity in feces and a reduction in the oxidation of linoleic acid. Trans-petroselinic acid, the trans isomer of petroselinic acid and an isomer of oleic acid, was shown in a study to increase cellular levels of triacylglycerols (TG) and cholesterol (TC) esters at a concentration of 100 μM. This also up-regulated the transcription of genes involved in fatty acid synthesis, including sterol regulatory element binding protein-1c (SREBP-1c), acetyl-CoA carboxylase alpha (ACACA), fatty acid synthase (FASN), stearoyl-CoA desaturase-1 (SCD1), 3-hydroxy-3-methylglutaryl-CoA reductase (HMGCR), 3-hydroxy-3-methylglutaryl-CoA synthase-1 (HMGCS1), farnesyl-diphosphate farnesyltransferase 1 (FDFT1), and SREBP-2. These effects were observed in human hepatoma HepG2 cells [56]. Our results indicate that *L. plantarum* P470 significantly decreased the level of trans-petroselinic acid, likely due to reduced fatty acid oxidation. These findings suggest that *L. plantarum* P470 can modulate lipid metabolism and generate short-chain fatty acids or other metabolites, leading to enhanced antioxidant and anti-inflammatory properties in fecal samples. This effect is primarily attributed to the regulation of the composition of intestinal microbiota.

However, our study has several limitations: significant individual differences, a small sample size leading to considerable intra-group variability, and suboptimal statistical analysis. Additionally, the fecal fermentation supernatant from healthy individuals did not exhibit significant antioxidant or anti-inflammatory activity, although there was a trend toward improvement in the overall fermentation supernatant. This suggests differences in fecal microecology and metabolic status between healthy participants and patients with CHD, which warrant further investigation with larger sample sizes. Factors such as patient gender, age, and geographical location should be considered to enhance the applicability of the results. Future research should explore a range of variables, including different diets, lifestyles, stages of disease, dosages, and timing of interventions, and should include larger, population-based cohort studies to confirm these findings.

## 5. Conclusions

To summarize, we examined the antioxidant and anti-inflammatory properties of *L. plantarum* P470, isolated from fermented Chinese Chives, utilizing an in vitro anaerobic fermentation model. Our findings suggest that *L. plantarum* P470 can increase hydroxyl radical scavenging activity and lower the expression of TNF-α and IL-6 in CHD feces. Furthermore, the *L. plantarum* P470 could be metabolized into SCFAs, which can regulate lipid metabolism by CHD gut microbiota. *L. plantarum* P470 was also found to decrease the F/B, as well as the abundance of other harmful bacteria such as *Escherichia-Shigella*, *Enterobacter*, *Veillonella*, *Eggerthella*, and *Helicobacter*, while increasing the abundance of beneficial genera such as *Bacteroides*, *Lactobacillus*, in the gut microbiota of CHD patient feces. This suggests that traditional fermented food such as chives can be used as a good strategy to regulate the intestinal microbiota to combat oxidation, inflammation, and slow down the progression of disease, especially by increasing the production of SCFAs. Overall, it is necessary to develop and utilize the effective components of traditional fermented food prebiotics and probiotics, which have significant potential in healthcare research.

## Figures and Tables

**Table 1:** Inflammatory factor-related primer sequence.

Gene	Oligonucleotide Sequence (5′–3′)
TNF-α	GGACTAGCCAGGAGGGAGAA
	CGCGGATCATGCTTTCTGTG
IL-6	TGGAGTACCATAGCTACCTGGA
	TCTCTCTGAAGGACTCTGGCT
IL-10	GGGCGAGTGTAACAAGACCT
	ATTTGCTGGGTTCCCACACT
β-actin	AGGGAAATCGTGCGTGACAT
	GGAAAAGAGCCTCAGGGCAT

**Table 2:** Different metabolite information of each group.

**CHD-48 vs. CHD-48-LP**									
**Compound**	**Formula**	**Molecular Weight**	**RT (min)**	**HMDB**	**PubChem**	**KEGG**	***p*** **-Value**	**Fold Change**	**log2(FC)**
Stearic acid	C_18_H_36_O_2_	284.27186	16.997	HMDB0000827	5281	C01530	0.044816	2.277	1.1871
Toxopyrimidine	C_6_H_9_N_3_O	139.07474	19.621	METPA0166	-	C01279	0.014444	0.15151	−2.7225
Geranylacetone	C_13_H_22_O	194.16701	17.101	HMDB0031846	1713001	C13297	0.012086	0.49681	−1.0092
Linoleate	C_18_H_32_O_2_	280.24017	16.121	HMDB0000673	5280450	C01595	0.0081258	0.14277	−2.8082
Inspra	C_24_H_30_O_6_	414.20414	10.885	HMDB0014838	443872	C12512	0.014593	4.0497	2.0178
A-12(13)-EpODE	C_18_H_30_O_3_	294.21941	16.398	HMDB0010200	16061061		0.026316	5.2634	2.396
α-linolenoyl Ethanolamide	C_20_H_35_NO_2_	321.2668	16.105	NA	NA	NA	0.034482	0.24847	−2.0089
**HP-48 vs. HP-48-LP**									
**Compound**	**Formula**	**Molecular Weight**	**RT (min)**	**HMDB**	**PubChem**	**KEGG**	***p*** **-value**	**Fold Change**	**log2(FC)**
L-aspartic acid	C_4_H_7_N O_4_	133.03669	0.772	HMDB0000191	5960	C00049	0.03759	0.077347	−3.6925
Dibutyl phthalate	C_16_H_22_O_4_	278.15181	17.236	HMDB0033244	3026	C14214	0.029904	2.6541	1.4082
D-(+)-malic acid	C_4_H_6_O_5_	134.02082	0.783	HMDB0031518	92824	C00497	0.036464	0.21635	−2.2086
2-methylcitric acid	C_7_H_10_O_7_	206.04345	0.847	HMDB0000379	5290	C02225	0.046553	0.059527	−4.0703
Linoleic acid	C_18_H_32_O_2_	280.24047	18.049	HMDB0000673	5280450	C01595	0.012485	0.17687	−2.4992
L-aspartic acid	C_4_H_7_NO_4_	133.03669	0.772	HMDB0000191	5960	C00049	0.038565	0.034422	−4.8605
Chenodeoxycholic acid	C_24_H_40_O_4_	438.29865	7.765	HMDB0000637	22833540	C05466	0.031712	26.812	4.7448
N-acetylneuraminic acid	C_11_H_19_NO_9_	309.10584	1.004	HMDB0000230	445063	C19910	0.049937	0.24727	−2.0159
Verapamil	C_27_H_38_N_2_O_4_	454.28316	6.051	HMDB0001850	2520	C07188	0.015041	3.1745	1.6665

## Data Availability

All data and materials are available from the corresponding author upon reasonable request.

## Supplementary Materials

The following supporting information can be downloaded at: https://www.mdpi.com/article/10.3390/nu16172945/s1, Table S1: The relative abundance at the phylum level; Table S2: The relative abundance at the family level; Table S3: The relative abundance at the genus level.
