# Supplementary material for: Lactiplantibacillus plantarum P470 Isolated from Fermented Chinese Chives Has the Potential to Improve In Vitro the Intestinal Microbiota and Biological Activity in Feces of Coronary Heart Disease (CHD) Patients
Source: Nutrients. 2024 Sep 2;16(17):2945. doi: 10.3390/nu16172945 (PMC11397641; doi:10.3390/nu16172945)
Supplement: Supplementary file 1 [file nutrients-16-02945-s001.zip › nutrients-3151302-supplementary.pdf]

**Table S1 The relative abundance at the phylum level**

| | HP-48(%) | HP-48-LP(%) | CHD-48(%) | CHD-48-LP(%) |
|---|---|---|---|---|
| Proteobacteria | 62.07±13.96 | 53.67±13.42 | 55.91±7.71 | 52.77±27.99 |
| Firmicutes | 18.37±11.98 | 28.98±24.88 | 22.73±12.89 | 25.69±27.89 |
| Bacteroidota | 15.19±13.17 | 14.45±16.09 | 13.34±14.96 | 17.18±17.15 |
| Actinobacteriota | 1.85±2.72 | 1.48±2.4 | 7.77±11.57 | 3.29±3.35 |
| Firmicutes/Bacteroidota | 2.35±1.85 | 2.00±1.54 | 1.76±0.86 | 1.49±1.62 |
| others | 2.20±2.43 | 1.42±0.91 | 0.24±0.17 | 1.06±0.78 |

**Table S2 The relative abundance at the family level**

| | HP-48(%) | HP-48-LP(%) | CHD-48(%) | CHD-48-LP(%) |
|---|---|---|---|---|
| Enterobacteriaceae | 43.75±23.49 | 24.31±24.7 | 50.47±8.33 | 39.75±28.03 |
| Pseudomonadaceae | 14.92±16.84 | 24.14±27.46 | 2.66±2.00 | 9.07±11.68 |
| Lactobacillaceae | 1.2±0.89 | 12.54±18.35* | 0.15±0.12 | 3.28±3.9* |
| Clostridiaceae | 0.33±0.26 | 0.26±0.48 | 5.2±9.68 | 9.31±18.53 |
| Bacteroidaceae | 9.08±13.15 | 10.15±15.84 | 8.71±8.88 | 13.32±15.81 |
| Eggerthellaceae | 0.32±0.44 | 0.12±0.19 | 3.89±7.69 | 0.28±0.4* |
| Veillonellaceae | 0.35±0.6 | 0.05±0.06* | 3.81±7.51 | 1.19±2.16 |
| Erysipelatoclostridiaceae | 0.42±0.34 | 0.25±0.33 | 0.76±0.92 | 3.31±6.46* |
| Acidaminococcaceae | 3.22±6.16 | 0.59±1.08* | 0.03±0.02 | 0.01±0.00 |
| Tannerellaceae | 1.98±2.35 | 1.04±1.57 | 4.09±5.46 | 2.18±2.76 |
| Lachnospiraceae | 4.5±3.73 | 4.01±4.22 | 2.24±1.8 | 2.14±2.07 |
| Peptostreptococcaceae | 0.15±0.05 | 0.3±0.34* | 1.53±2.00 | 2.58±4.58 |
| Enterococcaceae | 0.06±0.06 | 0.03±0.04 | 3.71±4.42 | 1.23±1.92 |
| Bifidobacteriaceae | 1.24±2.26 | 0.85±1.54 | 3.53±3.92 | 0.85±0.26 |
| Alcaligenaceae | 1.43±2.01 | 2.29±4.12 | 2.14±1.62 | 2.33±4.05 |
| Ruminococcaceae | 1.42±0.84 | 1.69±1.88 | 2.26±3.67 | 0.75±1.11 |
| Muribaculaceae | 1.75±2.87 | 1.81±3.27 | 0.12±0.23 | 0.15±0.19 |
| Micrococcaceae | 0.03±0.04 | 0.03±0.01 | 0.04±0.05 | 1.83±3.03 |
| Prevotellaceae | 1.42±2.03 | 0.83±1.6 | 0.35±0.56 | 1.42±2.84* |
| Oscillospiraceae | 1.56±2.17 | 0.9±1.41 | 0.11±0.09 | 0.24±0.3 |
| Comamonadaceae | 0.67±1.05 | 1.25±1.78 | 0.27±0.21 | 0.58±0.64* |
| Streptococcaceae | 0.88±1.63 | 0.09±0.07* | 0.57±0.64 | 0.34±0.56 |
| Burkholderiaceae | 0.13±0.13 | 0.88±1.52* | 0.04±0.05 | 0.25±0.3* |
| Listeriaceae | 0.77±1.53 | 0±0 | 0±0 | 0.01±0.02 |
| Clostridia_UCG-014 | 0.43±0.73 | 0.77±1.42 | 0.43±0.82 | 0.32±0.64 |
| Helicobacteraceae | 0.72±1.42 | 0.06±0.12* | 0±0 | 0±0.01 |
| Erysipelotrichaceae | 0.33±0.52 | 0.22±0.27 | 1.41±1.58 | 0.61±0.96* |
| Saccharimonadaceae | 0.36±0.46 | 0.59±0.95 | 0.09±0.11 | 0.07±0.14 |
| Bacillaceae | 0.53±0.98 | 0.11±0.21 | 0.17±0.3 | 0.04±0.08 |

| | | | | |
|---|---|---|---|---|
| Xanthomonadaceae | 0.51±0.87 | 0.19±0.24 | 0.17±0.2 | 0.33±0.5 |
| Others | 5.56±4.49 | 9.65±10.76 | 1.05±0.52 | 2.22±1.56 |

**Table S3 The relative abundance at the genus level**

| | HP-48(%) | HP-48-LP(%) | CHD-48(%) | CHD-48-LP(%) |
|---|---|---|---|---|
| Escherichia-Shigella | 41.62±22.23 | 22.54±26.25 | 42.04±13.93 | 27.91±12.53 |
| Pseudomonas | 14.92±16.84 | 24.14±27.46* | 2.66±2 | 9.07±11.68 |
| Lactobacillus | 1.2±0.89 | 12.54±18.35* | 0.15±0.12 | 3.28±3.9* |
| Clostridium_sensu_stricto_1 | 0.33±0.26 | 0.26±0.48 | 5.2±9.68 | 9.31±18.53 |
| Bacteroides | 9.08±13.15 | 10.15±15.84 | 8.71±8.88 | 13.32±15.81 |
| Klebsiella | 0.13±0.15 | 1.48±2.68* | 2.36±3.69 | 9.39±16.02 |
| Enterobacter | 0.09±0.1 | 0±0.01* | 4.66±9.12 | 0.33±0.63* |
| Eggerthella | 0.27±0.42 | 0.02±0.02* | 3.88±7.69 | 0.27±0.41* |
| Veillonella | 0.3±0.54 | 0.01±0.01* | 3.8±7.52 | 1.11±2.21 |
| Erysipelotrichaceae_UCG-003 | 0.24±0.43 | 0.09±0.1 | 0.54±1.01 | 3.27±6.45* |
| Parabacteroides | 1.98±2.35 | 1.04±1.57 | 4.09±5.46 | 2.18±2.76 |
| Acidaminococcus | 2.67±5.31* | 0±0 | 0.02±0.02 | 0±0* |
| Enterococcus | 0.06±0.06 | 0.03±0.04 | 3.71±4.42 | 1.23±1.92 |
| Bifidobacterium | 1.24±2.26 | 0.85±1.54 | 3.52±3.92 | 0.85±0.26 |
| Achromobacter | 1.43±2.01 | 2.29±4.12 | 2.14±1.62 | 2.33±4.05 |
| Romboutsia | 0.1±0.04 | 0.14±0.21 | 1.06±1.93 | 2±3.95 |
| Subdoligranulum | 0.4±0.59 | 0.73±1.19 | 1.89±3.65 | 0.35±0.61* |
| Muribaculaceae | 1.73±2.84 | 1.8±3.25 | 0.12±0.23 | 0.15±0.18 |
| Prevotella | 0.27±0.24 | 0.11±0.17 | 0.22±0.38 | 1.1±2.18* |
| Pseudarthrobacter | 0±0.01 | 0.03±0.01 | 0.01±0.03 | 1.14±1.95 |
| Blautia | 0.39±0.49 | 1.02±1.76 | 0.88±1.52 | 0.49±0.81 |
| Delftia | 0.04±0.04 | 0.95±1.79 | 0.08±0.08 | 0.34±0.56 |
| Streptococcus | 0.88±1.63 | 0.09±0.07* | 0.57±0.64 | 0.34±0.56 |
| Ralstonia | 0.1±0.11 | 0.86±1.52* | 0.04±0.05 | 0.25±0.3* |
| Listeria | 0.77±1.53 | 0±0 | 0±0 | 0.01±0.02 |
| Clostridia_UCG-014 | 0.43±0.73 | 0.77±1.42 | 0.43±0.82 | 0.32±0.64 |
| Helicobacter | 0.72±1.42 | 0.06±0.12* | 0±0 | 0±0.01 |
| Holdemanella | 0.02±0.02 | 0±0* | 0.72±1.28 | 0.11±0.13* |
| Ruminococcus_torques_group | 0.05±0.07 | 0.07±0.06 | 0.13±0.2 | 0.81±1.04 |
| Phascolarctobacterium | 0.55±0.85 | 0.59±1.08 | 0.01±0.01 | 0.01±0 |
| Others | 18.01±12.76 | 17.32±11.13 | 6.35±0.64 | 8.73±5.12 |
